# Nose-to-brain drug delivery: from bench to bedside

**DOI:** 10.1186/s40035-025-00481-w

**Published:** 2025-05-19

**Authors:** Isabell Drath, Franziska Richter, Malte Feja

**Affiliations:** 1 Department of Pharmacology, Toxicology and Pharmacy, University of Veterinary Medicine Hannover, Bünteweg 17, 30559 Hannover, Germany; 2 Center for Systems Neuroscience (ZSN), Hannover, Germany

**Keywords:** Intranasal, N2B, Parkinson's, Alzheimer's, Neurodegenerative disease, Nanoparticle

## Abstract

There is increasing interest in nose-to-brain delivery as an innovative drug delivery strategy for neurodegenerative disorders such as Parkinson’s or Alzheimer’s disease. The unique anatomy of the nose-brain interface facilitates direct drug transport via the olfactory and trigeminal pathways to the brain, bypassing the blood–brain barrier. Different administration techniques as well as advanced drug formulations like targeted nanoparticles and thermoresponsive systems have been explored to improve the delivery efficiency and the therapeutic efficacy. This review provides an up-to-date perspective on this fast-developing field, and discusses different studies on safety and pharmacokinetic properties. A thorough evaluation of preclinical and clinical studies reveals both promises and challenges of this delivery method, highlighting approved drugs for the treatment of epilepsy and migraine that successfully utilize intranasal routes. The current landscape of research on nose-to-brain delivery is critically discussed, and a rationale is provided for ongoing research to optimize therapeutic strategies.

## Background

The blood–brain barrier poses a challenge for the treatment of central nervous system (CNS) disorders by preventing most therapeutic agents from reaching the brain after oral or parenteral administration. Recently, intranasal administration of drugs has gained increasing interest as it can bypass the blood–brain barrier. This route is suitable for daily application and allows therapeutic molecules to be transported directly into the brain, bypassing the blood–brain barrier and increasing drug concentrations in the CNS [1]. Although the exact mechanisms of transport from the nose to the brain are not fully understood, preclinical and clinical studies have shown that the nose-to-brain drug delivery is applicable in both animals and humans. Here we summarize the current state of knowledge regarding mechanisms of nose-to-brain drug delivery including clinical applications.

## Anatomy and histology of the nose-brain interface

The nasal cavity of humans and other mammals including mice and rats is separated by the nasal septum into two parts. The two nostrils build the entrance to the nasal vestibule which merges into the nasal cavity. Within the nasal cavity there are three nasal conches, which form the nasal meatuses [2]. The whole nasal cavity except the nasal vestibule is coated with nasal mucosa which can be divided into the respiratory and the olfactory regions.

The respiratory region, which represents the major part of the nasal cavity, is coated with respiratory ciliated epithelium and serves primarily as a protective surface. It humidifies and warms the inhaled air and is able to remove particles and allergens [3]. Innervation of the respiratory mucosa is provided by branches of the trigeminal nerve [4].

**Fig. 1:**
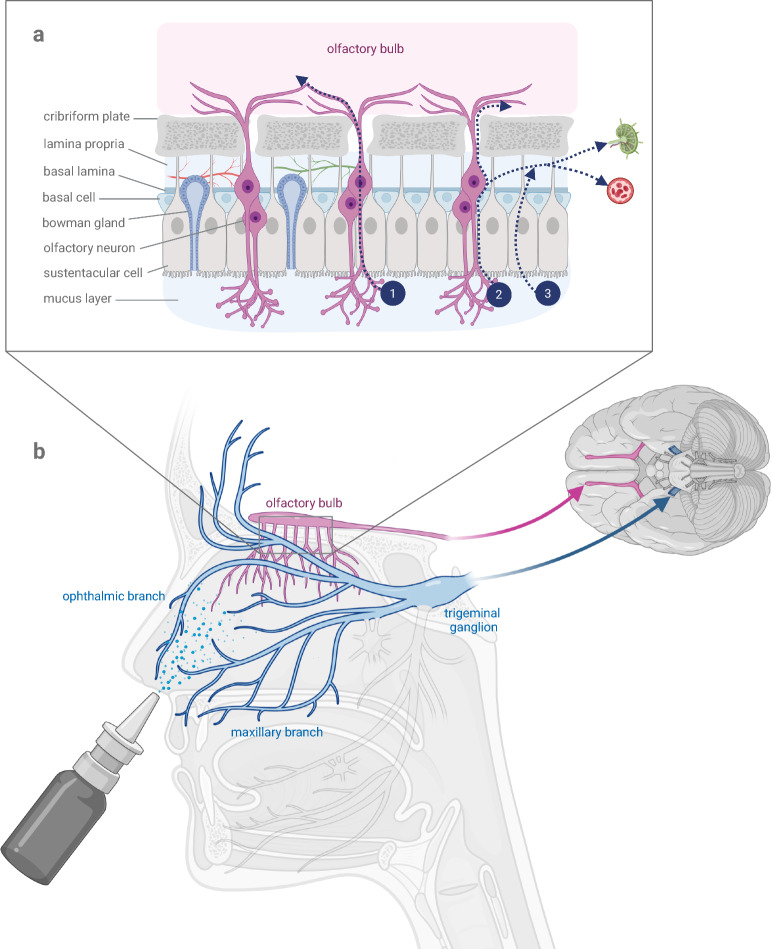
**a** General anatomy of the nose-to-brain interface and potential transport pathways for nose-to-brain delivery. The olfactory epithelium is composed of bipolar olfactory neurons, sustentacular cells, Bowman's glands, basal cells and its underlying lamina propria, which contains blood and lymph vessels. Axonal processes of olfactory neurons are arranged in bundles known as the fila olfactoria, which traverse the cribriform plate and reach the olfactory bulb. Potential pathways for drug delivery from the olfactory mucosa to the brain are illustrated in dark blue. ➀ Intracellular pathway: drugs are transported within olfactory neurons via axonal transport and endocytosis. ➁ Extra/paracellular transport along cranial nerves: drugs are transported within the perineural space, either to the cerebrospinal fluid in the subarachnoid space, or to the lamina propria, and subsequently to the blood or lymph vessels. ➂ Transcellular transport: drugs are transported through cells to the lamina proria with further transport to lymphatic vessels that are connected with the cervical lymph nodes or to blood vessels following entry to the systemic circulation. **b** Innervation of the nasal region by the olfactory nerve and branches of the trigeminal nerve. The olfactory nerve bundles, originating from the olfactory bulb, traverse the cribriform plate and provide innervation to the olfactory region of the nasal mucosa. The trigeminal nerve leaves the brainstem at the level of the pons and divides after the trigeminal ganglion into its three main divisions: V_1_, ophthalmic nerve; V_2_, maxillary nerve; V_3_, mandibular nerve. Only V_1_ and V_2_ send branches to the nasal epithelium, thereby innervating the respiratory mucosa and thus participating in the process of nose-to-brain delivery

The significantly smaller olfactory region is located at the roof of the dorsal meatus and is characterized by olfactory mucosa. Fine nerve fibers, the fila olfactoria*,* innervate the olfactory mucosa. They originate from the olfactory nerve, which innervates from the olfactory bulb through the lamina cribrosa to the olfactory region (Fig. 1a). Thus, the olfactory nerve displays a direct connection between the brain and the nose, which could mediate the higher efficiency of nose-to-brain delivery via the olfactory mucosa compared to the respiratory mucosa [5]. In addition to the sensory innervation by the olfactory nerve, the nose is sensitively innervated by branches of the trigeminal nerve. The trigeminal nerve originates at the pons, swells to form the ganglion trigeminale and divides afterwards into three main branches: nervus ophthalmicus, nervus maxillaris and nervus mandibularis (Fig. 1b). The ophthalmic and the maxillary nerves are responsible for the innervation of the nasal mucosa [6]. Moreover, the perineural space surrounding the cranial nerves is connected to the subarachnoid space and thereby directly connected to the cerebrospinal fluid (CSF) [7–9]. One pathway of CSF drainage is along the cranial nerves into the nasal epithelium [7, 10]. Taken together, the nose is directly connected to the brain and the CSF via the trigeminal and the olfactory nerves.

Another distinct characteristic of the two regions is the capillary density, which is about 5-times higher in the respiratory region than in the olfactory region [11]. High vascular density efficiently eliminates drugs from the tissue; therefore, low vascular density of the olfactory region correlates with higher brain delivery efficiency [11]. Furthermore, it is important to note that the olfactory epithelium in rats and mice is about 40%–50% of the total nasal surface; however, in humans the percentage is less than 10% [12–14]. This anatomical difference has a significant impact on the translation of preclinical studies from laboratory animals to humans, particularly for dose finding and the investigation of nasal delivery devices.

The olfactory mucosa consists mainly of four different cell types: ciliated olfactory receptors, supporting cells, basal cells and microvillar cells [15]. Ciliated olfactory cells are bipolar neurons, with one process that terminates in the olfactory bulb and another superficial process ending in a ciliated apical extension called olfactory vesicle [16]. Supporting cells, also called sustentacular cells, are non-ciliated epithelial cells, which function as metabolic support and are able to introduce substances into the surface mucus as well as to remove substances from it [17]. Basal cells are stem cells which do not reach the surface and are able to differentiate into ciliated olfactory receptors or supporting cells to replace degenerated cells [17]. Microvillar cells serve as bipolar sensory neurons [18]. The olfactory mucosa lies above the lamina propria which consists of blood and lymph vessels, Bowman's glands, nerve bundles and connective tissue [15].

The nasal blood supply is mainly provided by the external and the internal carotid arteries. The nasal walls are supplied by the sphenopalatine artery which comes from the maxillary artery, a branch of the external carotid artery. The anterior part of the nose is supplied with blood by the anterior and posterior ethmoidal arteries. These are branches of the ophthalmic artery, which originates from the internal carotid artery. Additionally, branches from the facial artery supply the vestibule and the anterior portion of the nose [19].

The human nose has several functions regarding breathing, which include air conditioning, heating of the inhaled air as well as protective mechanisms like mucociliary activity to remove particles and pathogens. The human average mucociliary transport rate is about 6 mm per minute [20] and especially particles over 15 μm in size are removed [19]. This mucociliary clearance mechanism reduces the retention time of drugs in the nose, which needs to be taken into account when developing intranasal drugs. Moreover, the human nose serves as a resonating body for speaking and is critical for the olfactory sense.

## Nose-to-brain transport routes

Nose-to-brain drug delivery is a promising avenue to circumvent the blood–brain barrier. Indications for a direct connection between the nose and the CNS were discovered at the beginning of the last century [8], as researchers were able to show that substances injected into the subarachnoid space can reach the nasal mucosa [21]. Numorous studies have substantiated this direct communication. However, the nose-to-brain pathway is still not completely understood and there appear to be different routes for drugs to reach the brain and distribute across its parenchyma (Fig. 1). Currently, two different routes of transport to the brain are proposed: the olfactory and the trigeminal routes. This concept is based on studies detecting high amounts of drugs in the lateral olfactory tract, olfactory bulb and trigeminal region after intranasal application [22–24].

First evidence for the olfactory route emerged almost 100 years ago when researchers suspected that viruses travel from the olfactory epithelium of the nose along the olfactory nerve to the brain [25]. This route of virus spread was brought back into focus of research by the COVID-19 pandemic [26]. Furthermore, latest research shows pathology in the olfactory bulbs after intranasal administration of alpha-synuclein preformed fibrils, which are thought to contribute to neurodegeneration in PD, underlining the connection between the nose and the olfactory part of the brain [27]. The olfactory route is subdivided into two pathways: (1) via the olfactory nerve to the olfactory bulb with subsequent parenchymal distribution to different brain areas and (2) distribution via the CSF followed by entrance into the brain parenchyma [28].

Early studies administering wheat germ agglutinin-horseradish peroxidase intranasally found high concentrations in the olfactory nerve and the glomerular layer of the olfactory bulb [29, 30]. Intranasally administered labeled siRNA distributed along the olfactory pathway to the brain and was detected within the olfactory epithelium, the olfactory nerve as well as in the glomerular and mitral cell layer of the olfactory bulb [31]. In detail, substances enter olfactory dendrites and are transported via the intracellular pathway across olfactory neurons into the fila olfactoria [32], subsequently reaching the olfactory bulbs by endocytosis and axonal transport [29, 33]. The axonal transport velocity has been shown to be at maximum 130 mm per day, thus it would take a minimum of 45 min for substances to reach the brain of a mouse after intranasal application [34]. Given that several membrane barriers need to be crossed, transcellular transport is generally expected to require more time compared to extracellular transport.

By measuring drug concentrations in the CSF after intranasal application, previous studies confirmed the second branch of the olfactory route: a direct pathway from the nose to the CSF [35–37]. It was previously shown that substances injected into the CSF would drain not only through the arachnoid villi, but also along the cranial nerves [38, 39]. This pathway is bidirectional [8], supporting the assumption of a direct pathway from the nose to the CSF. Substances are most likely transported extracellularly via olfactory lymphatic, perivascular and perineural spaces to the CSF [40]. This route is assumed to be a direct transport, as model predictions of the time required for different transport pathways to reach the olfactory bulb suggest that only convective bulk flow processes are fast enough to account for experimentally observed data [14]. A pharmacokinetic study showed that the maximum concentration in the CSF can be reached only 5 min after intranasal phenytoin application; such rapid transport favours a direct route from the nose to the CSF [41].

The trigeminal route includes transport along different branches of the trigeminal nerve that innervate the nasal respiratory mucosa: the ethmoidal nerve originating from the ophthalmic nerve, as well as the posterior nasal branches and the nasopalatinal nerve originating from the maxillary nerve [42, 43]. They are projecting to the trigeminal ganglion and to trigeminal nuclei in the brain stem. Furthermore, there is evidence that some trigeminal ganglion cells with sensory endings in the nasal epithelium have direct branches reaching into the olfactory bulb [44, 45]. Studies have shown that substances can reach the trigeminal nerve after intranasal application [46]. More precisely, a GLP-2 derivative was detected in the trigeminal principal sensory nucleus (Pr5) in the pons three minutes after intranasal administration, accounting for a rapid intracellular axonal transport [47]. In another study, insulin was shown to reach the perineural space of the trigeminal nerve, providing evidence that extracellular transport processes are also involved in the trigeminal pathway [48].

As any transport of drugs across barriers, the nose-to-brain delivery route is dependent on particle size and formulation of the substance administered. However, small size does not necessarily improve neuronal transport. For instance, 520-nm Poly(lactic-co-glycolic acid) nanoparticles can be detected in neuronal bundles after intranasal administration, indicating their transcellular neuronal transport, whereas 80-nm and 175-nm particles were only detected in other cell types, albeit with a more rapid distribution [49]. Moreover, neuropeptides like GLP-1 and GLP-2 derivatives with functional sequences were shown to be rapidly transported through trigeminal axons after intranasal application in mice [47, 50]. Others reported that the trigeminal pathway serves as the dominant route for intact nanoparticles, whereas the olfactory pathway is more likely to deliver substances which are already released from nanoparticles [51].

Of note, substances transported predominantly via the olfactory route rapidly appear in different brain areas, suggesting extracellular transport to reach the brain and the CSF [52]. In contrast, substances transported mainly via the trigeminal pathway take longer to reach the brain, indicating that they are more likely to be transported transcellularly [47]. Moreover, the trigeminal nerve is longer than the olfactory nerve, leading to longer transport duration.

## Techniques for intranasal administration

An optimal technique for intranasal application is crucial for successful delivery of drugs to the brain. Several different techniques and devices have been developed for nose-to-brain delivery, including ultrasound-mediated methods [53], electric guidance of charged particles [54] and catheter-based administration [55]. In preclinical studies using small laboratory animals, micropipettes are frequently used to place small droplets at the entrance of the nostrils to be breathed in by the animal. In order to define factors that impact delivery efficacy, influences of animal position, body weight and age were investigated [56]. Interestingly, older animals with higher body weights require increased intranasal dosages to reach the same drug concentration in the brain as in young animals. Furthermore, placement of animals in a supine or an upright position has no effect on the delivery efficiency [56]. To further enhance delivery efficiency and precision, catheters are applied to specifically target the olfactory region of the nasal cavity in mice and rats [55, 57]. The catheter reduces untargeted distribution to the periphery compared with the standard pipette-based method. However, the procedure is more invasive and requires anesthesia.

Another method to enhance delivery efficiency is the use of external magnetic fields to guide charged particles to the olfactory region. Electric guidance reduces particle loss in the anterior nose and increases particle deposition in the olfactory region [54]. With the use of a magnetic field and certain improvements in particle diameter and injection angle, the olfactory deposition in an in silico study of the human nose was 65-times higher compared to standard injections without magnetic fields [58]. This indicates that improvement of application methodology and the use of external magnetic fields can enhance nose-to-brain delivery.

One disadvantage of the above described techniques for nose-to-brain drug delivery is that drugs are delivered to the entire brain unspecifically, whereas most CNS diseases primarily affect certain brain regions or cell types. Thus, concentrated drug application to the target cells could enhance efficacy and reduce off-target effects. The combination of intranasal delivery and focused ultrasound with microbubbles has been suggested as a targeted strategy [53, 59]; however, this method can be quite disruptive for brain tissue and its use may be limited to severe conditions such as certain brain tumors. In addition to brain region targeting, it is also possible to use selective cell-targeting nanoparticles to deliver drugs to disease-related cells. For example, in glioblastoma therapy, it is of great importance to target cancer cells while reducing exposure of healthy cells. Co-layering nanoparticles with poly-l-glutamate and hyaluronate increases glioblastoma targeting in tumor-bearing mice after intracranial injection [60]. Targeting dopaminergic neurons could be promising in the field of Parkinson's disease (PD), whereas targeting amyloid plaques may be an interesting therapeutic approach in Alzheimer's disease (AD) [61]. Modified silica nanoparticles with dopamine ligands that bind to neuronal dopamine receptors showed superior delivery of glutathione to SH-SY5Y cells compared to unmodified nanoparticles, as well as improved cytoprotective and anti-apoptotic effects in vitro [62]. Furthermore, a study has demonstrated neuron-selective delivery of microRNA using a D3-peptide-conjugated nanopolymer injected into the tail vein in an AD mouse model [63]. Cationic siRNA complexes have also been shown to precisely target amyloid plaques in the brains of AD mice after intravenous injection [64]. Whether such selective cell-targeting can be combined with nose-to-brain delivery remains to be determined.

**Fig. 2:**
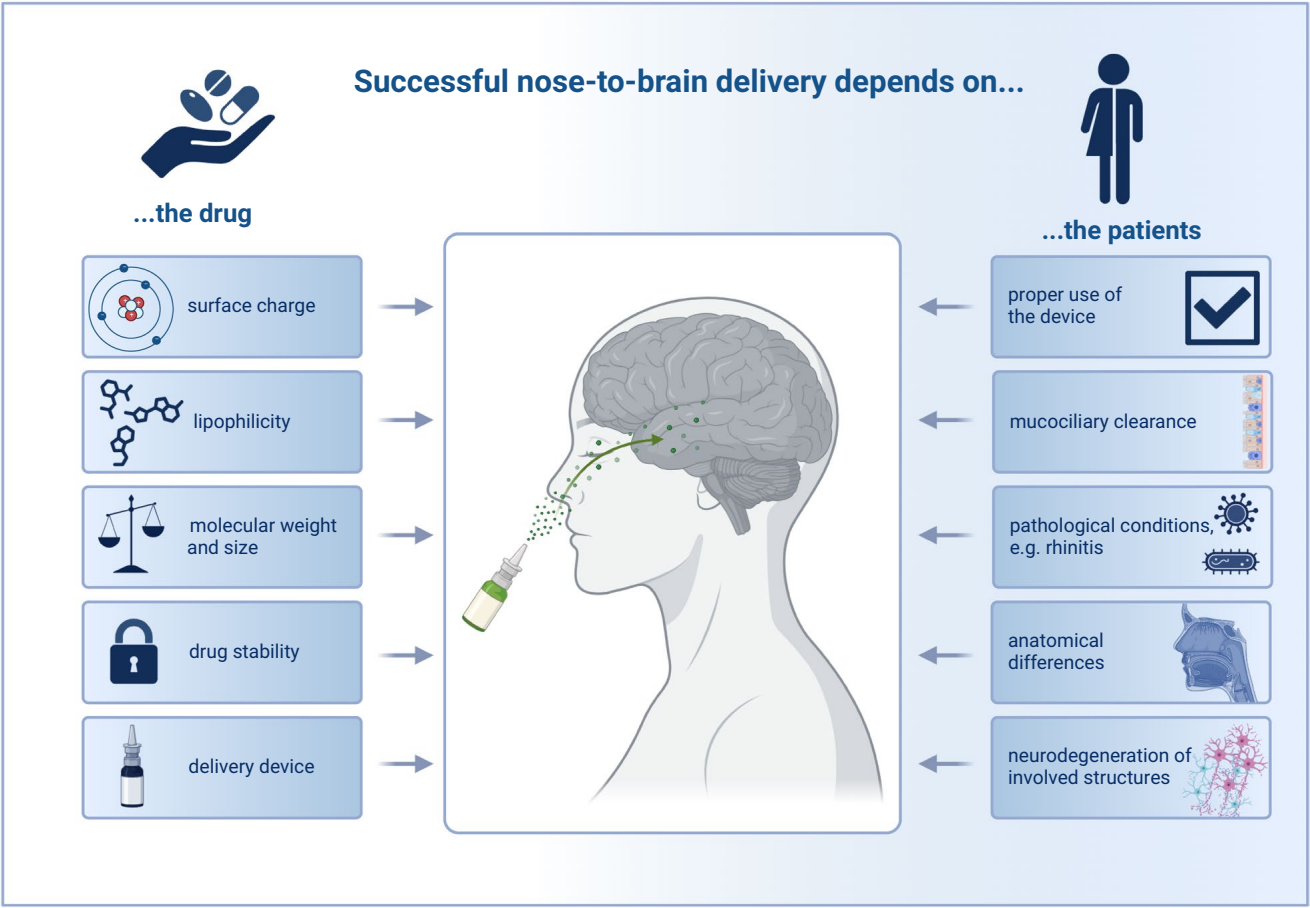
Factors influencing nose-to-brain delivery

Another challenge for nose-to-brain drug delivery is the development of appropriate delivery devices, since standard devices are optimized for local effects and therefore deliver only a small amount of drug to the olfactory region [65]. For instance, traditional spray pumps deliver only around 5% of the drug to the upper nasal space where the olfactory mucosa is located [66]. The development of intranasal drugs with appropriate delivery devices is limited by the fact that their use in preclinical studies cannot be accomplished with the same delivery devices as in humans because of the different size and anatomy of laboratory animals. Mathematical models and human nasal replica casts are used to overcome this species barrier; however, successfull delivery in clinical studies remains challenging [67] (Fig. 2). For instance, four different nasal spray pumps and four nasal nebulizers failed to deliver a therapeutically significant amount of certain particles to the olfactory region [65]. Therefore, it is argued that nasal replica casts most times do not imitate a human nose sufficiently to draw valid conclusions for a regulatory drug deposition study [68]. Despite these challenges, there are several different nasal delivery devices on the market, some of which are specifically designed for nose-to-brain delivery. One such device uses a propellant-powered delivery technology which claims to reduce the amount of drugs that get trapped in the nasal vestibule, thereby delivering more medication to the upper part of the nose compared to traditional nasal sprays [69]. This device has been used in clinical studies to deliver dihydroergotamine mesylate for acute treatment of migraine attacks and has shown rapid pain relief as well as good tolerability [70, 71].

Further developments in the field are bidirectional breath-powered delivery devices [72], one of which has already been approved for migraine treatment with sumatriptan nasal powder. The device consists of an exhalation mouthpiece connected to the nosepiece by the device body. During exhalation the soft palate closes and seperates the nasal from the oral cavity, which prevents unintentional delivery to the oropharynx or lungs. The breath-powered delivery devices deliver more drugs to the target site with a better pharmacokinetic profile than traditional nasal sprays [73, 74].

## Pharmacokinetics of nose-to-brain drug delivery

The goal of pharmacokinetic studies for nose-to-brain drug delivery is to develop the optimal drug formulation, determine dosing regimens, and gain understanding of drug interactions. Previous pharmacokinetic studies have shown that the peak concentrations of substances like clomipramine nanoparticles [75], dantrolene [76] or donepezil liposomes [77] in the brains of rodents can be reached as early as 20–30 min after intranasal application. In contrast, another study measured the highest concentration of intranasally administered nanoparticles containing fibroblast growth factor after 30 min only in the olfactory bulb, while the peak concentration in the rest of the rat brain was reached 4 h after application [78]. Further studies generated pharmacokinetic profiles of intranasal vascular endothelial growth factor in rats and intranasal allopregnanolone in mice, which measured the highest drug concentrations in the trigeminal nerve, optic nerve and olfactory bulb followed by the cerebrum and hippocampus [79, 80]. The lowest concentrations in the brain were measured in the cerebellum and even lower concentrations in the serum after intranasal application of fibroblast growth factor in rats [81]. Due to a good vascularization of the nasal mucosa, drugs are reaching the systemic circulation. In addition to different brain areas, drugs like methotrexate reach even the cervical lymph nodes of rats with peak concentrations one hour after intranasal administration [79, 82]. Different studies have revealed superiority of the nasal route compared to other administration routes. Intranasal administration of a hematopoietic growth factor, which has limited capacity to cross the blood–brain barrier, is 8–12 times more effective than subcutaneous application in brain and CSF delivery [83]. Furthermore, a recent study demonstrated that the brain uptake of human recombinant erythropoietin, curcumin, glucagon-like peptide 1 and anti-Aβ antibodies given to mice by intranasal administration is more than 5 times higher than that by intraperitoneal injection [84]. The superior efficiency of brain delivery by intranasal administration compared to intraperitoneal administration has also been observed for a peptide capable of Aβ hydrolysis [85]. Compared to oral administration, the nasal route achieved 25-fold higher bioavailability of harmine nanocrystals in the brain [86]. Moreover, intranasal applications achieved higher, longer-lasting brain drug concentrations compared to intravenous administration [81, 87]. For instance, the area under curve for carmustine concentration in the brain after intranasal administration was 14.7 times that of intravenous administration [88]. Furthermore, oxytocin, as well as high-molecular-weight substances such as cobrotoxin, reach the brain in greater amounts when administered intranasally than when administered intravenously [89, 90].

Differences in pharmacokinetic profiles are apparent between experimental animals and humans. After intranasal administration of insulin lispro in humans as well as in dogs, the insulin lispro was detectable in the CSF of dogs, while its level in the CSF was below the limit of quantification in humans [91]. A computational model comparing intranasal delivery patterns between mice and humans has been developed for further pharmacokinetic studies [92]. The model predicted that nasally administered nanomaterials reach the mouse brain at an amount of two orders of magnitude compared to that reaching the human brain. This suggests that extrapolation of pharmacokinetic studies from laboratory animals to humans is of limited validity and should always be done with caution.

## Drug formulations for nose-to-brain delivery

Another key aspect in drug discovery is finding an appropriate drug formulation that delivers the drug in a stable, safe and effective manner (Fig. 2). For nose-to-brain administration, drugs can be formulated as powders, solutions or gels. Furthermore, biochemical features like particle size, pH and charge can be adjusted. By optimizing the formulation, it is possible to enhance brain uptake by more than 10 folds [93].

As stated above, particle size is an important determinant of neuronal uptake. Furthermore, particles of different sizes also distribute differently inside the nasal cavity after application, which can be addressed by optimizing the formulation. In a 3D model of human nose, microparticles with a size of 10 μm reach the olfactory region at a higher amount than particles with a size of 2 μm [94]. The maximum olfactory deposition was observed with particles of 8–12 μm in size [95]. In line with these findings, another study reported that inertial 10-μm particles and diffusive 1-nm particles have higher olfactory deposition than particles in the size range of 10 nm–2 μm, probably due to the inertial force of 10-μm particles and the Brownian motion of 1-nm particles [96]. This is consistent with another study that found highest olfactory deposition for very small particles with a size of 1–2 nm [97].

To avoid nasal irritation and achieve efficient drug absorption from nasal mucosa, it is important to adjust the pH of the formulation [98]. While the pH level of the nasal mucosa is approximately 6.3 [99], studies have revealed a higher absorption of nasal formulations at pH below 4.79 [100]. Furthermore, to prevent irritation and maintain microbial defense, the pH of the nasal formulation should be adjusted slightly acidic, as lysozymes in nasal secretions effectively destroy bacteria at acidic pH but become inactivated under alkaline conditions, leaving tissue vulnerable to infection [101]. Considering the advantages of an acidic pH and the physiological environment of the nose, intranasal formulations should ideally have a pH level between 4.5 and 6.5, which is important to avoid adverse effects on the mucosa or the ciliary movement [102].

In addition, lipophilicity, molecular weight and surface charge of intranasal drugs also affect the delivery efficiency. Increasing lipophilicity is correlated with higher drug concentrations in the CSF after intranasal administration [103]. Furthermore, decreased molecular weight of drugs is associated with higher drug concentrations in the CSF [104]. Anionic drug carriers provide a 20% increase in drug targeting efficiency compared to cationic carriers [105].

### Formulations in gel, solution or powder?

One obvious disadvantage of solutions is the short residence time in the nasal cavity. Gels with higher viscosity remain longer at the mucosa and deliver significantly higher brain concentrations of the drug compared to solutions [106, 107]. To prolong the residency time of gels further and overcome rapid clearance of the drug, gels that respond to temperature, ions or pH with higher viscosity are being developed [108, 109]. A thermo-responsive gel turning viscous at 32 °C not only increases drug concentrations in the brain compared to a non-thermo-responsive gel, but also sustains the concentrations for a longer period of time [110]. Besides, it is possible to add mucoadhesive agents to the formulation, which can improve biodistribution by increasing the retention time in the brain and the brain/blood ratio [111]. In most cases, either solutions or gels were used, but a small number of studies also investigated powder formulations. Some studies showed superiority of powders, while others achieved better delivery results using other formulations, depending on several factors, such as the drug administered. For *L*-3,4-dihydroxyphenylalanine (*L*-DOPA) administration in PD patients, powders appear to act more rapidly than solutions, which may be an advantage depending on the treatment goal [112]. Two different nasal powders applied with an active delivery device provide direct transport rates over 60% [113]. In contrast, other studies showed superiority of eutectic formulations compared to powders [114].

### Nanoparticle-mediated nose-to-brain delivery

However, efficient delivery to the brain is challenging, and whether using powders, sprays, solutions or gels, vectors are commonly used in the treatment of CNS diseases. Notably, in the field of neurodegenerative diseases, adeno-associated viruses are the most commonly used carriers. However, they have some disadvantages, such as limited loading capacity, difficult vector production and inflammatory reactions [115–117]. Non-viral vectors avoid these disadvantages and are safer for patients. For this reason, several studies have explored non-viral carriers, such as liposomes or nanoparticles to enhance brain bioavailability of various drugs. It has already been demonstrated that nanoscaled carriers support the transport of substances to the brain and at the same time increase the stability of active ingredients [118]. Lipid nanoparticles loaded with paclitaxel and miltefosine improved drug concentration in the mouse brain by 5 folds compared to the free drug [118]. A substantial number of studies have indicated superiority of nanoparticle-mediated delivery versus plain delivery in terms of pharmacokinetic parameters as well as treatment efficacy [119–121]. There is a wide range of materials that can be used to create nanoparticles. Materials should be carefully selected depending on the purpose. Nanoparticles should reach the brain and penetrate target cells while being non-toxic to the nasal mucosa or the brain. Meanwhile, they may have a direct effect on the efficacy of the drug. For instance, tyrosine modification on nanoparticles for siRNA therapeutics improves the siRNA-mediated knockdown efficacy [122]. Whether nanoparticles have a branched or a linear structure also makes a difference to biocompatibility [122, 123]. Moreover, a meta-analysis reported that lipid nanoparticles are significantly superior to polymeric nanoparticles in enhancing the brain bioavailability of drugs [124]. In addition, the coating of nanoparticles can also influence their properties: chitosan coating appears to improve mucoaffinity and diffusion efficiency in vitro [125]. In conclusion, there are several ways to modify nanoparticles and improve nose-to-brain delivery of a particular drug.

## Safety considerations for nose-to-brain delivery

Safety is a fundamental aspect that needs to be addressed when developing drugs towards regulatory approval. In the development of intranasal drugs it is important that not only the drug itself but also the excipients of the drug formulation like mucoadhesives or nanoparticles are safe and do not cause any side effects. Regarding nose-to-brain delivery it is important to consider systemic side effects as well as local nasal mucosa and CNS toxicity.

A major advantage of intranasal compared to systemic delivery is the limited amount of drug reaching the systemic circulation and the liver [126], thereby reducing the risk of systemic adverse effects and rapid metabolization [127]. Different studies have confirmed general safety of intranasal drugs or have shown even higher safety compared to other administration routes. For instance, clinical studies testing intranasal recombinant erythropoietin recorded only mild adverse events without severe adverse events. Moreover, the number of adverse events in the treatment group was not increased compared to the placebo group [128, 129]. Furthermore, intranasal delivery of paliperidone palmitate, a drug which causes serious adverse events after oral administration, did not cause any alterations in blood parameters in rats, suggesting intranasal delivery as a promising tool to reduce systemic side effects [130].

Due to the direct contact of the drug with the nasal mucosa, it is very important to screen for local toxic effects and to consider different factors influencing nasal conditions such as temperature, humidity or conditions like rhinitis. Previous evaluation of cytotoxicity to the nasal mucosa was done in cell culture models of nasal mucosa, mainly including primary cells collected from the olfactory region of rats [131] and the immortalized cell line RPMI 2650 isolated from a squamous cell carcinoma of the nasal septum [132]. These models can be used to carry out cytotoxicity assays as well as permeation studies, e.g., measurement of transepithelial electrical resistance [133]. The nasal tissue has protective functions which, among others, include mucociliary activity to prevent exogenous particles reaching the upper airways. Thus, it is essential to ensure that the drug has no adverse effects on the ciliary movement. To this purpose, ciliotoxicity ex vivo studies on sheep nasal mucosa were conducted, monitoring for ciliary or epithelial damage, necrosis or hemorrhage in response to nanoparticle-mediated drug delivery [77]. Interestingly, nanoparticle formulation may decrease local nasal toxicity and improve safety at effective dosages. In rats, mucosa irritation was assessed after intranasal administration of free-diazepam solution compared with an aqua-triggered in-situ gelling microemulsion containing diazepam. While the free-diazepam led to mild to moderate histopathological mucosal lesions, the gelling microemulsion left the mucosa intact [107]. Thus, if a drug is known to cause local mucosal irritation, it may be possible to find carriers that can deliver the drug without local side effects [134].

Besides the adverse effects on mucosa and cilia, it is crucial to ensure that there are no effects on the olfactory nerve and the CNS. To predict effects on neuronal cells, in vitro assays using neuroblastoma cell lines such as SH-SY5Y cells or primary neuronal cells, can be utilized [135, 136]. Lactate dehydrogenase, MTS (3-(4,5-dimethylthiazol-2-yl)-5-(3-carboxymethoxyphenyl)-2-(4-sulfophenyl)-2H-tetrazolium) or MTT (3-(4,5-dimethylthiazol-2-yl)-2,5-diphenyl-2H-tetrazolium bromide) assays can be employed to assess cell viability and cytotoxicity in response to different drugs. More advanced models would also include models of blood–brain barrier penetration, like transwell or microfluidic models [137]. In addition, computational models could be used to predict CNS side effects [138]. General health and potential adverse effects in the CNS can be preclinically assessed in laboratory animals using the modified Irwin screen or the functional observation battery [139]. Moreover, preclinical evaluation includes neurotoxicity studies in rodents using histopathological evaluation of brain and nerve tissue to check markers for neuroinflammation or cytotoxicity. Neuroinflammation and neurotoxicity could lead to deficits in cognition or olfaction [140, 141]. Nonclinical CNS safety assessments of chronic nose-to-brain drug delivery should therefore incorporate evaluations of cognitive and olfactory functions in laboratory animals, as the olfactory bulb and prefrontal cortex, which play key roles in olfaction and cognition, respectively, are anatomically proximal to the intranasal administration site. Relevant behavioral tests include the buried food or olfactory habituation/dishabituation tests for assessing olfactory function [142], as well as tasks probing executive functions such as decision-making and inhibitory control (delay discounting, five-choice serial reaction time task) [143, 144], working memory (delayed alternation task) [145], attention (attentional set-shifting task) [146], and behavioral flexibility (reversal learning task) [147], which are heavily dependent on prefrontal cortex activity.

## Clinical relevance of nose-to-brain delivery and pre-clinical studies

Neurodegenerative diseases are one of the leading causes of death worldwide with an increasing prevalence in the aging society [148]. Although first slight improvements were shown for pathology-targeting antibody therapy, there is a lack of efficient disease-modifying treatment options due to incomplete knowledge of the pathophysiology and the challenges to overcome the blood–brain barrier. Similarly, treatment of debilitating neurological diseases such as epilepsy or migraine is hampered by insufficient drug levels in the brain and by side effects. Despite the success in preclinical studies, translation of nose-to-brain efficacy to the clinics remains challenging. Nevertheless, over the last twenty years there has been an increasing number of clinical studies evaluating nose-to-brain delivery as a novel option for the treatment of neurodegenerative and neurological diseases. The following sections will summarize current pre-clinical drug development and clinical trials, as well as successfull applications for nose-to-brain drug delivery.

### Drug development for nose-to-brain delivery for PD

**Fig. 3:**
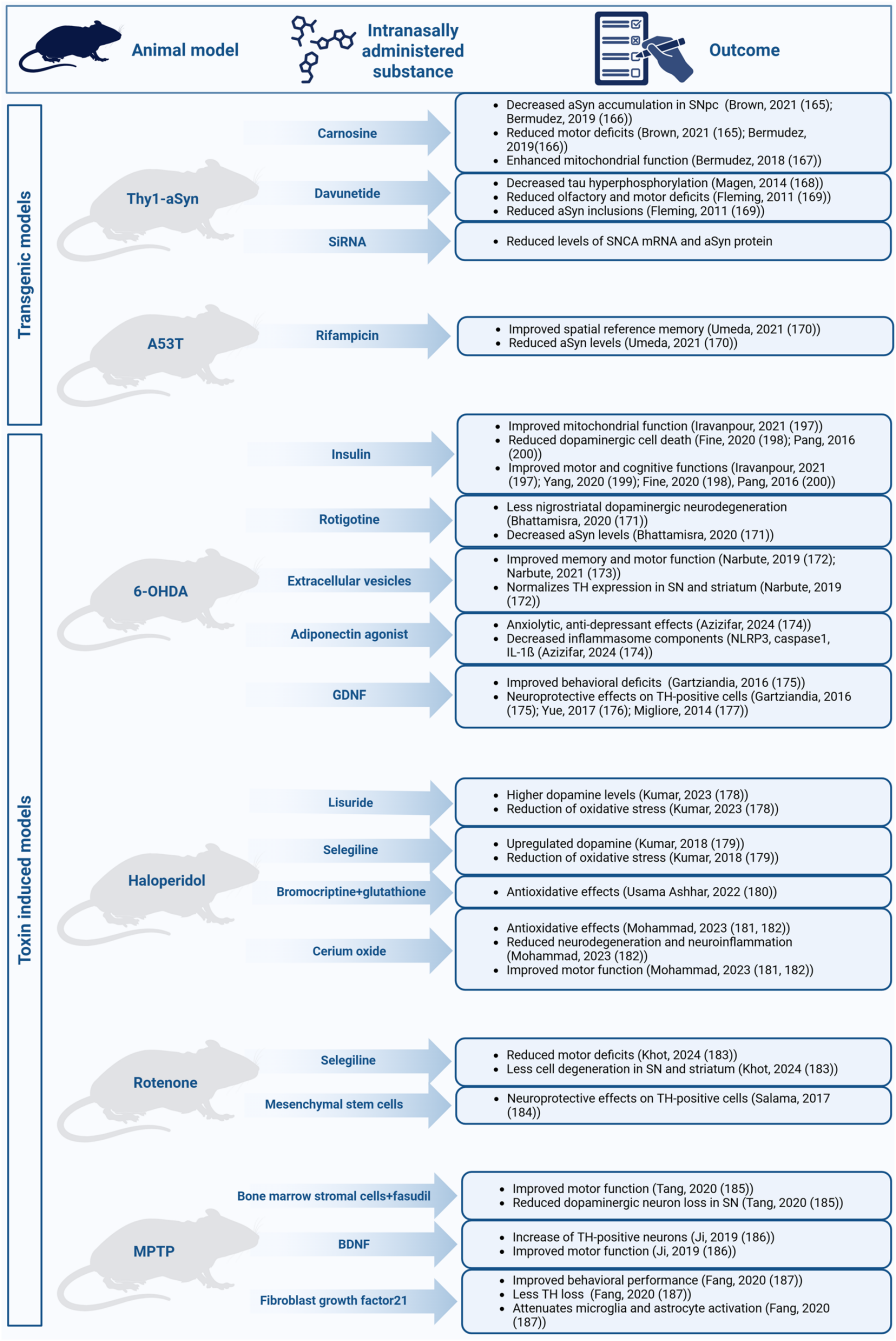
Preclinical studies using nose-to-brain drug delivery in different rodent models of PD. The Thy1-aSyn mouse model overexpresses human alpha-synuclein under the Thy1 promoter, while the A53T mouse model overexpresses the A53T-mutant human alpha-synuclein under the mouse prion protein promoter. Models generated by injection of different toxins include 6-hydroxydopamine (6-OHDA), which is a synthetic monoaminergic neurotoxin. Haloperidol is an antipsychotic medication that has been observed to induce parkinsonism as an adverse effect. Rotenone is an isoflavonoid that is typically utilized as an insecticide and acaricide. 1-methyl-4-phenyl-1,2,3,6-tetrahydropyridine (MPTP) is a neurotoxin that selectively targets dopaminergic neurons

PD is the second most common neurodegenerative disease and the most common movement disorder worldwide, affecting more than 1% of the population over the age of 60 [149–151]. In addition to the classic motor symptoms such as rigor, tremor and postural instability, patients also show non-motor symptoms like cognitive deficits, anxiety and gastrointestinal dysfunction [152, 153]. Symptoms like nausea, dysphagia and delayed gastric emptying make alternatives to oral treatment even more necessary [154, 155]. The symptoms are based on a complex pathology that includes degeneration of dopaminergic neurons in the substantia nigra [156] and a striatal loss of dopamine [157], as well as accumulation and aggregation of alpha-synuclein [158], the main component of Lewy bodies [159], in various regions of the brain [160]. Current PD therapy is limited exclusively to improving hypokinetic, motor symptoms with dopamine substitutes, such as *L*-DOPA, and therefore improves the quality of life of patients for several years, but also leads to the development of uncontrolled, involuntary movements, known as dyskinesia [161, 162]. In addition, this therapy cannot address many motor (e.g., changes in speech) and non-motor disturbances (e.g., memory loss, anxiety disorders, gastrointestinal dysfunctions), or prevent or halt the progressive loss of dopaminergic neurons. Thus, there is no disease-modifying treatment for PD, even though the number of patients continues to increase [152, 163]. There are different treatment approaches in the pipeline of drug development, with some focusing on further improvement of dopamine replacement, while others targeting alpha-synuclein-related pathology [164]. In the following section, we point out some examples for preclinical (Fig. 3) and clinical studies (Table 1) with intranasal delivery for treatment of PD.

**Table 1:** Clinical studies using nose-to-brain drug delivery for the treatment of PD

Study title	Treatment	Study number (NCT)	Study Phase	Subject number	Status	Location	Related publication
Treatment of Parkinson Disease and Multiple System Atrophy Using Intranasal Insulin	Insulin vs. placebo	NCT02064166	2	15	Completed	Worcester, Massachusetts, United States, University of Massachusetts Medical School	[205]
The Effect of Intranasal Insulin on Motor and Non-motor Symptoms in Parkinson’s Disease Patients	Insulin vs. placebo	NCT04687878	2	40	Recruiting	Tehran, Iran, Shahid Beheshti University of Medical Sciences, Shohada-e-Tajrish Hospital	n.a
Intranasal Insulin in Parkinson’s Disease	Novolin R vs. placebo	NCT04251585	2	30	Recruiting	Saint Paul, Minnesota, United States, HealthPartners Neuroscience Center	n.a
CNS Uptake of Intranasal Glutathione	Reduced glutathione	NCT02324426	1	15	Completed	Seattle, Washington, United States, University of Washington	[210]
Intranasal Glutathione in Parkinson’s Disease	Glutathione vs. placebo	NCT01398748	1	34	Completed	Kenmore, Washington, United States, Bastyr Clinical Research Center	[209]
Phase IIb Study of Intranasal Glutathione in Parkinson’s Disease	Reduced glutathione 100 vs. 200 mg vs. placebo	NCT02424708	2	45	Completed	Kenmore, Washington, United States, Bastyr Clinical Research Center	[211]
						Seattle, Washington, United States, University of Washington	
Intranasal Insulin and Glutathione as an Add-On Therapy in Parkinson’s Disease	Novolin R + glutathion vs. matched placebo	NCT05266417	2	56	Recruiting	Davie, Florida, United States, Institute for Neuroimmune Medicine	n.a
Intranasal Human FGF-1 for Subjects With Parkinson’s Disease	Human FGF-1 450 vs. 900 µg	NCT05493462	1	4	Not Yet Recruiting	Nassau, Bahamas, The Medical Pavilion Bahamas	n.a
Therapeutic Potential for Intranasal Levodopa in Parkinson’s Disease -Off Reversal	*L*-dopa 35 mg vs. 70 mg vs. 140 mg vs. *L*-dopa 70 mg + carbidopa 70 mg vs. placebo	NCT03541356	2	32	Completed	Sydney, New South Wales, Australia, The Brain and Mind Centre/Scientia Clinical Research Brisbane; Queensland, Australia Q-Pharm Brisbane; Queensland, Australia The Mater Hospital Melbourne; Victoria, Australia, The Alfred Hospital Perth; Western Australia, Australia, Perron Institute	n.a
Tolerance to NeuroEPO in Parkinson Disease	NeuroEPO vs. placebo	NCT04110678	1/2	26	Completed	Havana, Cuba, Clinic of Movement Disorders, International Center for Neurological Restoration; La Habana, Cuba, Centro Immunologia Molecular CIM	[128, 215]
Parkinson’s Disease Therapy Using Cell Technology	Autologous mesenchymal stem cells vs. placebo	NCT04146519	2 + 3	50	Recruiting	Minsk, Belarus, The Belarusian Medical Academy of Postgraduate Education	[216]

Rotigotine is a dopamine agonist, which has promising potential for the treatment of PD [188], but its use is challenging due to the low bioavailability and high first-pass effects after oral administration, as well as application-site reactions after transdermal treatment [189]. Nose-to-brain delivery could be an efficient tool to improve the bioavailability of rotigotine. A pharmacokinetic study revealed that intranasal delivery of rotigotine-loaded nanoparticles achieves higher brain levels than intravenous rotigotine application [87]. In line with these results, another study showed higher tyrosine-hydroxylase signal in nigrostriatal dopaminergic neurons in the 6-hydroxydopamine (6-OHDA) rat model of PD after intranasal administration of lactoferrin-modified rotigotine nanoparticles [190].

First-line therapy for PD includes monoamine oxidase B inhibitors, such as selegiline, as they reduce metabolic degradation of dopamine and its replacements. However, only 10% of the oral selegiline dose is bioavailable, leading to the need of high, daily doses causing a long list of adverse effects [191]. Preclinical studies have shown higher bioavailability as well as reduced motor deficits after intranasal administration of selegiline in a rotenone PD rat model [192, 193]. Further, intranasal application of selegiline nanoemulsion has led to increased dopamine in the brains of rats [179].

Metabolic dysfunctions, including accumulation of lipids like polyunsaturated fatty acids and cholesterol, are involved in the misfolding and aggregation of alpha-synuclein [194]. In addition, abnormal binding of alpha-synuclein to oxidized lipid metabolites causes malfunction of mitochondria [195]. Accordingly, one approach to treating PD is targeting lipid metabolic abnormalities [196]. As antidiabetic drugs like insulin can regulate lipid metabolism, they have been explored in preclinical and clinical studies for PD. As mentioned before, insulin distributes along the trigeminal nerve and reaches the CNS after intranasal application in rats [14]. Several preclinical studies showed that intranasal insulin treatment leads to an improvement in mitochondrial functions as well as a reduction of dopaminergic cell death in a rat 6-OHDA model of PD [197, 198]. Intranasal insulin treatment also attenuates motor and cognitive deficits in the 6-OHDA rat model of PD [197–200].

With increasing attention paid to gene therapies, RNA interference has appeared as an interesting therapeutic approach to reducing alpha-synuclein and its downstream pathology. Small interfering RNAs (siRNAs) can be delivered to the brain more effectively via the nose than through the intravenous route [46]. In the alpha-synuclein-overexpressing Thy-1-aSyn mice [201, 202], intranasally administered siRNA-loaded polymeric nanoparticles are able to reach different brain regions including the substantia nigra, and significantly reduce *SNCA* mRNA expression as well as alpha-synuclein protein level in the brain [203]. Thus, intranasal administration using nanoparticles could provide a non-invasive route to chronically apply small nucleotides to the brain.

In PD, about half of the clinical trials using the intranasal drug delivery method focus on treatment with either insulin or glutathione, or a combination of both. Besides the beneficial effects of intranasal insulin in preclinical studies, a case study described a patient with manganese-induced parkinsonism whose motor and cognition symptoms improved after four weeks of intranasal treatment with insulin [204]. Furthermore, a clinical proof-of-concept study for intranasal insulin including 16 subjects with clinically diagnosed PD or multiple system atrophy [205] confirmed the safety and showed improvement of motor and cognitive symptoms. Other clinical trials in study phase II are running to test efficacy with an increased number of patients (Table 1).

Glutathione, which plays a protective role against oxidative stress, mitochondrial dysfunction and cell death [206, 207], is depleted in PD patients [208]. Clinical trials have been conducted to restore the glutathione levels in PD patients by delivering glutathione intranasally. Two different phase I studies have confirmed the safety and CNS uptake of glutathione after intranasal administration [209, 210]. The following phase IIb study showed symptomatic improvement after three months of intranasal administration, although after a wash-out period of four weeks, glutathione was not superior to placebo [211]. Of note, a survey revealed that over 86% of patients using glutathione nasal spray rated this route of administration as convenient and easy to use, supporting good compliance [212]. In the context of intranasal delivery of glutathione, the MAD Nasal™ mucosal atomization device (MAD; Teleflex, Morrisville, NC) has been used in a randomized, double-blind phase I/IIa study [209]. The MAD is a syringe equipped with a soft, conical nozzle that forms a seal with the nostril, thereby preventing expulsion of the drug. The liquid drug is atomized into particles ranging from 30 to 100 µm [213]. For clinical studies involving intranasal insulin, the ViaNase™ delivery device (Kurve Technology, Inc. Lynnwood, WA) has been used [205]. ViaNase™ is an electronic atomizer that nebulizes a metered dose of the drug into a chamber covering the patients nose. Patients then inhale the drug by breathing regularly for a predetermined time.

As shown in Table 1, there are further studies using various drugs for intranasal therapy in PD patients. For example, a study achieved positive effects on cognition by treating PD patients with intranasal recombinant human erythropoietin [214].

### Drug development for nose-to-brain delivery for AD

AD is the most common cause of dementia worldwide with an increasing prevalence in the aging society. AD has two hallmark pathologies: extracellular beta-amyloid plaques [217] and neurofibrillary tangles composed of hyperphosphorylated tau [218]. The most prominent clinical symptoms are progressive memory deterioration, disordered behavior, and impairments in language, comprehension and visual-spatial skills [219]. There is no cure for AD, and current treatment options are mainly limited to symptomatic management. Recently, the U.S. Food & Drug Administration (FDA) has approved application of plaque-reducing antibodies, which is hampered by severe side effects in a subgroup of patients.

An extensively studied approach for the treatment of AD is intranasal insulin, which is already being tested in clinical trials. Intranasal insulin restores the cerebral glucose metabolism and mitigates astroglial activation and neuronal loss in a streptozotocin-induced AD rat model [220]. Further, intranasal insulin in rats restored levels of different AD-related, dysregulated microRNAs and decreased tau phosphorylation, amyloid-beta aggregation and neuroinflammation [221]. Moreover, intranasal insulin ameliorated memory and learning deficits in AD rat models in different preclinical studies [221–223].

Furthermore, researchers aim to slow the progression of neurodegeneration. Several AD mouse models (cholinotoxin-induced, amyloid-beta-induced [224] and transgenic APP/PS1 mice) showed improvement of memory function and spatial cognition after receiving intranasal application of colivelin [225, 226], a synthetic derivate of humanin that plays a role in suppressing neuronal death [227]. Furthermore, intranasal application of basic fibroblast growth factor leads to improved memory in a rat model of AD [81].

The use of stem cell transplantation for the treatment of neurodegenerative diseases is very promising, albeit limited by the blood–brain barrier. Intranasal delivery could make it more feasible in the future. Repetitive intranasal application of human neural stem cells decreases neuroinflammation, and enhances neurogenesis and expression of beta-amyloid-degrading enzymes in a APP/PS1 mouse model of AD [228]. Also, intranasal administration of the secretome of cortical neural stem cells to 5 × FAD mice reduces amyloid-beta accumulation and ameliorates memory function [229]. Plaque reduction, alleviation of gliosis, and increased neuronal density in certain brain regions have been seen in an AD mouse model after intranasal delivery of mesenchymal stem cell secretome [230].

Intranasal siRNA application is also a promising approach for AD. Intranasal administration of siRNA targeting the β-site amyloid precursor protein cleaving enzyme 1 (*BACE1*) combined with rapamycin, an approved immunosuppressant, led to a reduction in amyloid-beta deposition and improvement of cognition in a transgenic AD mouse model [231]. Another preclinical study combined siRNA targeting *BACE1* and siRNA targeting caspase-3, to inhibit neuronal apoptosis in 3 × Tg-AD mice. They constructed lesion-recognizing siRNA-nanoparticles for intranasal administration. Results showed positive treatment effects on memory deficits in the 3 × Tg-AD mice [232].

Nose-to-brain delivery has been used in several clinical trials for AD over the last decades. A number of clinical studies have explored the potential of intranasal insulin application for AD, showing that insulin could reduce amyloid plaques and improve verbal memory [233, 234]. Past clinical trials employed various dosages, ranging from single-dose to long-term administration, and used different types of insulin, including fast-acting insulin aspart [235], fast-acting insulin glulisin [236], regular human insulin [237] and the long-acting analog insulin detemir [238]. In these studies, insulin aspart showed superiority to regular insulin in terms of treatment efficacy [235]. Several studies confirmed the safety of intranasal insulin in patients with mild cognitive impairment and AD [67, 239]. Recent evidence suggests that intranasal insulin treatment increases the volume of certain brain regions, which is associated with memory improvement [240]. Intranasal insulin also leads to changes in inflammatory markers in the CSF, suggesting that intranasal insulin may not only treat symptoms but also influence the progression of AD [241]. On the other hand, there are studies showing no significant treatment effects, which could be due to a small number of subjects [239] or due to inadequate delivery devices [67]. Pilot studies on intranasal insulin in AD patients and older adults at high dementia risk using the previously mentioned ViaNase™ for drug delivery, showed sufficient insulin penetration into the brain via CSF analyses [237, 242]. Nevertheless, the trial-specific modified device demonstrated insufficient reliability in a clinical study, necessitating its mid-trial replacement with the Precision Olfactory Delivery (POD®) device (Impel Pharmaceuticals, Seattle, WA) [67]. The POD® uses a liquid fluorocarbon propellant to inject a metered dose of liquids or powders to the olfactory epithelium without electronic assistance [69]. In addition, clinical trials are currently ongoing for intranasal stem cell treatment of AD. As mentioned above, preclinical studies in this field have shown promising results. The challenge is now to transfer these findings from preclinical to clinical feasibility. Two studies investigating intranasal administration of bone marrow-derived stem cells in 100 participants (NCT03724136) and 500 participants (NCT02795052), respectively, are currently in progress and expected to be completed in July 2025*.*

### Drug development for nose-to-brain delivery for epilepsy

Epilepsy is a chronic neurological disorder characterized by recurrence of seizures of central origin. Those seizures are the result of the interaction between pathological excitation and a lack of inhibition in the neuronal networks of the CNS. Current antiseizure medications do not cure epilepsy. They merely manage seizures and must be taken continuously, leading to lifelong exposure of the entire body to the drug, necessitating high safety standards. Although various antiseizure medications have been approved and most patients respond well to the therapy, about 30% of patients do not become seizure-free under currently available treatment [243, 244]. One possible cause is the insufficient drug levels in the brain. Therefore, strategies like acute and chronic intracerebral microinfusion via drug pumps were tested in a rat seizure model [244, 245]. Even though the studies showed beneficial effects in preclinical models, it is important to consider alternative, less invasive delivery routes. Non-invasive approaches such as nose-to-brain delivery may help achieve higher drug levels in the CNS while minimizing systemic exposure [126]*.* Another challenge in the treatment of epilepsy is to find an optimal application route to stop acute seizures. Emergency treatment in epilepsy patients is generally administered orally or intravenously, if the patient can swallow during the seizure or if a medically-trained person is available to inject intravenously. Therefore, intranasal emergency treatment could be an option to ease the administration especially in a non-medical setting at home and thereby accelerate treatment [246].

To maximize the effects of lamotrigine, a seizure-suppressing substance approved for oral application, researchers developed lamotrigine-containing nanocapsules or nanoparticles for intranasal use. By now, there are a few studies on the pharmacokinetics and bioavailability of intranasal lamotrigine in rats and mice, showing promising results with high brain-targeting efficacy [247, 248], but further research has to be done especially regarding efficacy. Another orally-administered seizure-suppressing drug carbamazepine has also been tested for intranasal delivery. Intranasal application of a mucoadhesive carbamazepine nanoemulgel to pentylenetetrazole-treated mice delayed the onset of convulsion and death compared to intravenously-injected animals [249]. The use of neuropeptides is another approach in the treatment of epilepsy. Intranasal treatment with nanoparticles containing thyrotropin-releasing hormone significantly reduced the seizure afterdischarge duration and increased the number of stimulations required to reach a generalized tonic–clonic seizure in a kindling model of temporal lobe epilepsy [250].

Benzodiazepines, including lorazepam, diazepam, and midazolam, are the most commonly used substances for acute seizure management. When administered intranasally in rats, a thermosensitive gel containing lorazepam-loaded nanostructured lipid carriers, reduced the prevalence of pentylenetetrazole-induced seizures by two-thirds and decreased the severity and duration of symptoms compared to the sham group [251]. Moreover, intranasal mucaoadhesive clobazam microemulsion showed enhanced brain uptake in a pentylenetetrazole-induced mouse model compared to intravenous clobazam, leading to a faster increase in seizure threshold [111].

**Fig. 4:**
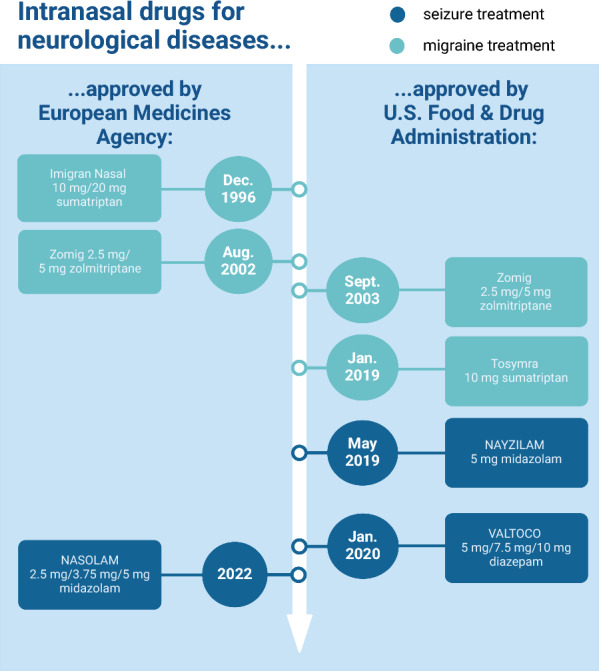
Approved drugs using nose-to-brain delivery for the treatment of neurological diseases

Based on the promising preclinical studies, intranasal benzodiazepines were tested in human patients and approved for the treatment of acute seizures (Fig. 4). The first FDA-approved nasal treatments for acute seizures are midazolam (NAYZILAM) and diazepam (VALTOCO) nasal sprays [252]. In line with this, the European Medicines Agency (EMA) approved midazolam nasal spray (NASOLAM) in 2022 [253]. A retrospective study confirmed the efficacy and safety of midazolam nasal spray in humans [254], indicating it as a promising application route. For intranasal application in acute seizure management, it is important that the delivery device is user-friendly and does not require any active participation from the patient. Consequently, conventional unidose nasal sprays are commonly used in non-professional settings, whereas MADs are typically used by healthcare professionals for emergency treatment [255, 256].

### Drug development for nose-to-brain delivery for migraine

Migraine is an episodic form of chronic headache that occurs in attacks, often accompanied by nausea and vomiting. There are different classifications of migraine by the International Headache Society Headache Classification Committee, such as migraine with or without aura [257]. An aura manifests itself, for example, in the form of very different neurological symptoms such as visual field defects, paresthesia and paresis [258, 259]. Migraine affects more than one billion people of all ages worldwide [260] and significantly reduces their quality of life with an impact on social life and work [261]. Women are three times more likely to be affected than men [262]. The pathophysiology is still not completely understood as it is a complex disorder of nervous system function including genetic causes and the influence of neuropeptides [263]. Therefore, treatment consists mainly of acute medication for pain relief, while preventive treatment is not very established. Oral application of triptans like sumatriptan, zolmitriptan and rizatriptan is used most frequently. Despite the established migraine treatment with oral triptans, there is a need for optimization of the route of administration. After oral administration, triptans need to be absorbed from the gastrointestinal tract to reach the blood circulation and have to cross the blood–brain barrier to get to the CNS. On the way to the brain, a high amount of the drug gets lost, through first-pass metabolism of the liver, leading to the fact that high doses are required. Migraine patients often have gastro-intestinal complaints such as diarrhea, nausea or vomiting, which can occur as a symptom of the migraine or as an adverse side effect of treatment [264]. Nevertheless, low oral bioavailability of these compounds makes alternative delivery options for brain targeting necessary. In the following, we highlight preclinical studies investigating nose-to-brain delivery of triptans as well as clinical studies that showed efficiency for approved intranasal migraine treatment.

Solid lipid nanoparticles of sumatriptan succinate optimized for brain targeting showed fast permeation across nasal mucosa in an ex vivo study using goat nasal mucosa, without altering the integrity of the mucosa [265]. Furthermore, a pharmacokinetic study in rats showed higher brain levels after intranasal delivery of lipid sumatriptan nanoparticles compared to intravenous application [266]. A sumatriptan-loaded nano-ethosomal mucoadhesive gel showed beneficial effects on behavioral as well as biochemical parameters in a nitroglycerin-induced migraine rat model [267]. Different pharmacokinetic studies in rats and mice demonstrated that zolmitriptan reaches the brain faster and in greater amounts when administered intranasally compared to intravenously [114, 268].

For the treatment of migraine, there are already approved triptan nasal sprays on the market (Fig. 4). Sumatriptan nasal spray was approved by the EMA in 1996 under the trading name “Imigran nasal” and by the FDA in 2019 under the name “Tosymra”. Sumatriptan nasal powder has a better outcome in the reduction of nausea compared to oral sumatriptan in human patients, indicating the advantages of nasal treatment [269]. Moreover, zolmitriptan was also approved by the EMA in 2002 and by the FDA in 2003 under the name “Zomig”. Zolmitriptan nasal spray is superior to zolmitriptan tablets, with rapid onset of headache relief (only 15 min after dosing) [270]. Although zolmitriptan nasal spray has already been approved, efforts are still being made to further optimize the marketed nasal spray by using different kinds of nanocarriers like chitosan nanoparticles, nanoethosomes or novasomes [268, 271, 272]. Proper drug delivery devices play a crucial role in ensuring effective treatment. Most approved triptan nasal sprays use Advaspray®, a Unidose liquid nasal spray (Aptar, Crystal Lake, IL), which is user-friendly but does not explicitly target the upper nasal space [213, 273]. To improve drug delivery to the upper nasal cavity, particularly the olfactory epithelium, advanced bidirectional breath-powered delivery devices are used. One such approved delivery system is ONZETRA® Xsail® (Currax Pharmaceuticals, Brentwood, TN), which administers sumatriptan nasal powder [274]. Besides enhanced deposition to the upper nasal space, oral exhalation during application closes the soft palate, thereby preventing lung deposition [275]. However, one drawback of these advanced devices is that they require a certain level of patient compliance and are less convenient to use than traditional nasal spray.

## Conclusion

Nose-to-brain delivery provides a promising option to circumvent the blood–brain barrier in a non-invasive route suitable for a long-term, repetitive and easy-to-apply regimen. Nasal application is proven to be safe or even enhance safety of drugs compared to systemic application in preclinical and clinical studies. Moreover, many studies have shown superior delivery efficacy of nasal applications compared to other application types. Nevertheless, the development of specific nasal delivery devices that target primarily the olfactory region for high delivery efficacy is challenging, currently limiting the application to potent drugs. Furthermore, a better understanding of the precise delivery routes and uptake mechanisms will enable improvement of strategies, such as optimizing carrier systems and targeting specific brain regions or cells. In conclusion, the nose-to-brain delivery is a promising, innovative form of application with enormous implications for treating CNS diseases.

## Data Availability

Data sharing is not applicable to this article as no datasets were generated or analysed during the current study.

## Abbreviations

6-OHDA: 6-Hydroxydopamine
AD: Alzheimer's disease
BACE1: β-Site amyloid precursor protein cleaving enzyme 1
BDNF: Brain-derived neurotrophic factor
CNS: Central nervous system
CSF: Cerebrospinal fluid
EMA: European medicines agency
FGF-1: Fibroblast growth factor 1
MAD: Mucosal atomization device
PD: Parkinson's disease
siRNA: Small interfering RNA
